# Supplementary material for: Effects of theophylline on ADCY5 activation—From cellular studies to improved therapeutic options for ADCY5-related dyskinesia patients
Source: PLoS One. 2023 Mar 3;18(3):e0282593. doi: 10.1371/journal.pone.0282593 (PMC9983822; doi:10.1371/journal.pone.0282593)
Supplement: S1 Appendix — (DOCX) [file pone.0282593.s001.docx]

**SUPPLEMENTARY INFORMATION**

**Effects of theophylline on ADCY5 activation -**

**From cellular studies to improved therapeutic options for ADCY5-related dyskinesia patientsl**

Dirk Tänzler^1,2^, Marc Kipping^1,2^, Marcell Lederer^3^, Wiebke F. Günther^1,2^, Christian Arlt^1,2^ Stefan Hüttelmaier^3^, Andreas Merkenschlager^4^ and Andrea Sinz^1,2^*

^1^ Department of Pharmaceutical Chemistry & Bioanalytics, Institute of Pharmacy, Martin Luther University Halle-Wittenberg, Halle (Saale), Saxony-Anhalt, Germany

^2^ Center for Structural Mass Spectrometry, Martin Luther University Halle-Wittenberg, Halle (Saale), Saxony-Anhalt, Germany

^3^ Institute of Molecular Medicine, Section for Molecular Cell Biology, Faculty of Medicine, Martin Luther University Halle-Wittenberg, Halle, Saxony-Anhalt, Germany

^4^ Department of Neuropediatrics, Hospital for Children and Adolescents, University of Leipzig, Leipzig, Saxony, Germany.

*Corresponding author

Email: [andrea.sinz@pharmazie.uni-halle.de](mailto:andrea.sinz@pharmazie.uni-halle.de) (AS)

The Supplementary Information contains the dosage scheme for theophylline (as slow-release formulation) and all LC/MS raw data underlying Figures 3 and 5. The folder names (Figure3A, Figure3BC, and Figure5) reflect the respective figure numbers in the main manuscript.

The files include:

- Raw data
- TargetLynx
- Excel files of quantitation analysis

Dosage of Theophylline (Slow-Release Formulation) for Treating Patients with ADCY5-Related Dyskinesia

The use of theophylline as a slow-release formulation offers a specific treatment of patients with ADCY5-related dyskinesia due to its mode of action at the adenosine A2A receptor. Theophylline has been shown to exhibit only minor side effects in children with status asthmaticus that were treated with a low-dose of theophylline (5-7 mg/kg per day). The relatively narrow therapeutic window of theophylline can be addressed by slowly increasing the dose of theophylline and by monitoring theophylline blood levels.

The following dosage scheme has been applied by us:

- A preschool-aged patient with ADCY5-related dyskinesia that had been successfully treated with 150 mg of caffeine (3x 50 mg daily) was treated with 400 mg of slow-release theophylline (2x 200 mg daily).

- Initially, theophylline and caffeine were given in combination, but during the course of the therapy, the daily caffeine dose was reduced over a period of five months until theophylline was administered alone.

- The theophylline dose was gradually increased from ~6 mg/kg/day to ~23 mg/kg/day over a time course of 8 weeks. Theophylline blood levels ranged between 7.5 mg/l to 21.1 mg/l.

- At a theophylline dose of ~12 mg/kg/day (divided into two single doses), the following effects were observed: The patient became more upright, showed increased muscle tone, and was able to stand and walk independently. The quality of sleep improved as dyskinetic movements subsided completely when falling asleep.

- By increasing the theophylline dose to ~23 mg/kg/day, continuous improvements were observed.

- We did not observe any adverse side-effects during theophylline dose titration. Arterial blood pressure, pulse rate, and theophylline-related laboratory parameters were within the given reference ranges.

- The following blood parameters should be regularly checked after administration of theophylline as slow-release formulation:

Theophylline

Electrolytes (Na, K)

Complete blood count

Creatinine

Urea

ASAT

ALAT

TSH (hyperthyreosis is possible)

Blood pressure should also be checked.
