# Supplementary material for: Effects of theophylline on ADCY5 activation—From cellular studies to improved therapeutic options for ADCY5-related dyskinesia patients
Source: PLoS One. 2023 Mar 3;18(3):e0282593. doi: 10.1371/journal.pone.0282593 (PMC9983822; doi:10.1371/journal.pone.0282593)
Supplement: Attachment — Submitted filename: Response_to_Reviewers.docx [file pone.0282593.s003.docx]

***Responses to Reviewer’s Comments***

*Question: In this paper, Tänzler et al. report the results of theophylline and caffeine administration on cAMP concentrations in WT and ADCY5 mutant cells, as well as the results of theophylline treatment in a ADCY5 patient. It is overall well-written and easy to read. Even though both the cellular data and clinical report are of interest, there are issues with the way data are interpreted and what the authors conclude.*

*Response:* We would like to thank this reviewer for his/her overall favorable comments on our work. I would like to point out that we performed all experiments in a diligent and careful manner and we believe that our conclusions are fully justified.

*Question: The first issue concerns the in vitro model that is used: both a non-neuronal (HEK) and non-integrated (cells) model. Given the complexity of striatal function, this limits greatly what can be inferred from the results – which should be mentioned in text.*

*Response:* We are aware that the cellular system used herein does not reflect the situation in the striatum. However, given the outstanding results in the patient described in this study (as well as in nine further patients treated with theophylline worldwide now) this impressive improvement of dyskinesia confirms the results of our cellular studies. We added a sentence addressing this issue in the “Conclusions” part on page 15: “We are aware that the cellular system used herein does not reflect the situation in the striatum. However, given the outstanding results in the patient described in this study - as well as in other patients treated with theophylline worldwide now - this impressive improvement of dyskinesia confirms the results of our cellular studies.”

*Question: The authors draw conclusions concerning caffeine and theophylline efficacy on cAMP lowering (“most prominent reduction of cAMP concentration for istradefylline, followed by theophylline and caffeine”, “strikingly, the reduction in cAMP level was more pronounced for theophylline compared to caffeine”) that are not supported by the data they present. Indeed, the same concentrations have been used for both molecules even though they are not equivalent, and the differences are clearly not statistically significant. This need to be rephrased, and limitations need to be clearly presented.*

*Response:* We agree with the reviewer that according to Figure 3B, the effects of cAMP reduction for caffeine and theophylline are similar to each other after 10 minutes of drug treatment. However, considering the effects of caffeine and theophylline at 60 minutes after drug treatment, the effect of theophylline in respect to cAMP level reduction is more pronounced at a concentration of 100 µM. We rephrased the respective sentences in the text: “The most prominent reduction of cAMP levels was observed for istradefylline, followed by theophylline and caffeine showing a less pronounced cAMP reduction.” “Strikingly, the reduction in cAMP levels was slightly more pronounced for theophylline compared to caffeine.”

*Question: The authors present theophylline as a safe treatment, choosing to cite a paper reporting only minor side effects but at very low doses (5-7 mg/kg per day), even though they treat their ADCY5 patient with 23 mg/kg per day, and they do not cite any report on theophylline toxicity. Yet therapeutic window is known to be extremely narrow with this molecule, and there are several reports of sometimes fatal theophylline toxicity on the literature. So much so that theophylline is not prescribed for asthma if other medications are available (*[*https://www.ncbi.nlm.nih.gov/books/NBK532962/*](https://www.ncbi.nlm.nih.gov/books/NBK532962/)*). Such toxicity is only reported with extremely high doses of caffeine, making it the safest treatment of the two, which should be said in text.*

*Response:* We are fully aware of the narrow therapeutic window of theophylline, which however can be efficiently met by a careful up-dosing of the drug. The reason why theophylline is not being used as first-line treatment for asthma bronchiale any more is that the development of ß_2_-sympathomimetics has advanced tremendously in the last 20 years. These drugs, such as salbutamol, salmeterol etc, exhibit a better efficacy for treating asthma bronchiale as theophylline.

During the last months, I have been approached by a number of parents of children suffering from ADCY5-related dyskinesia and I am advising neurologists and pediatricians on how to safely use theophylline. For this, I have assembled a guideline for medical doctors (and patients) regarding the dosage of theophylline as slow-release formulation for treating patients with ADCY5-related dyskinesia. We have now included this guideline in the Supporting Information of this manuscript.

Due to the spectacular success of theophylline treatment in the patient described in our manuscript who was confined to a wheelchair and is now able to walk without help, nine children with ADCY5-related dyskinesia are now being treated worldwide with theophylline. The videos the parents share with me are partially breathtaking. In none of the cases, side effects are observed for theophylline when the dosage is slowly increased as described in our dosage scheme and blood parameters are monitored regularly as described (see Supporting Information).

To clarify this important point, two sentences were added on pages 13, 14, and 15: “Theophylline exhibits a narrow therapeutic window, which however can be efficiently met by a careful up-dosing of the drug and by regularly monitoring relevant blood parameters (see Supporting Information).” “Several patients are now being treated worldwide with theophylline. In none of the cases, side effects are observed for theophylline when the dosage is slowly increased as described in our dosage scheme and blood parameters are monitored regularly as described (see Supporting Information).” “Theophylline, on the other hand, is available as a slow-release formulation, showing only minor side-effects, even in pediatric applications despite its narrow therapeutic window.”

*Question: What’s more, the authors sate that theophylline was more effective than caffeine, but they used comparatively much higher doses of theophylline than caffeine in this patient. Even though doses up to 800 mg/day have been used in the recent report cited by the authors, they seem to have stopped at 150 mg a day in their patient. Have higher doses been tried? Have side effects prevented to raise the dose? In all likelihood, the benefits observed from high-dose theophylline treatment would have been observed with higher doses of caffeine, so that concluding here to the superiority of theophylline is quite misleading (the only advantage that could be put forward here is its slow-release formulation). The case report part should therefore be rewritten to make room for all this additional (and crucial) information.*

*Response:* We assume that this reviewer is referring to the publication [Meneret A, Mohammad SS, Cif L, Doummar D, DeGusmao C, Anheim M, et al. Efficacy of Caffeine in ADCY5-Related Dyskinesia: A Retrospective Study. Mov Disord. 2022;37(6):1294-8. Epub 20220405. doi: 10.1002/mds.29006. PubMed PMID: 35384065] that we are also quoting in our manuscript (literature reference [14]). The reviewer is certainly aware that the caffeine dose of 800 mg/day was applied to a 17-year old male patient. Caffeine doses are however not given in mg/kg body weight, which is a deficiency of this publication. It seems obvious that the dose administered to a 17-year-old male patient has to be higher than that applied to a preschool-aged female patient. Therefore, a caffeine dose of 150 mg/day was absolutely sufficient for the patient described herein. Higher doses of caffeine have in fact been tried (up to 3 x 200 mg/day), but no improvement of dyskinesia symptoms was observed any more. Specifically, the patient was never able to walk under caffeine treatment, while under theophylline treatment the patient started walking independently.

The reviewer should also note that in that specific publication (Meneret et al, literature reference [14]) other examples are shown where caffeine was administered to a 15-year-old female patient at 100 mg/day with much improved symptoms and to a 13-year-old female patient at 225 mg/day with very much improved symptoms. This clearly demonstrates that the caffeine dose administered in the patient described herein is not too low.

*Question: The authors state in the conclusion that istradefylline exhibits severe side effects. What are their sources? Most reports seem to conclude to limited side effects and acceptable tolerance with this drug. Most reported side effects appear to be dyskinesia, confusion and hallucinations, which are common in a population of Parkinson’s disease patients but cannot be generalized to other patients. Therefore, this needs to be rephrased.*

*Response:* Istradefylline is currently being evaluated at the EMA for approval in the EU to treat Parkinson’s disease. It is known that istradefylline exhibits side effect that have so far hindered approval of this drug in the EU. It is not proven that the side effects of the drug can fully be attributed to common effects that are also observed in Parkinson’s patients. Nevertheless, the respective sentence was rephrased: ”As istradefylline exhibits side-effects even at low-dose application it is currently not an approved drug in Germany.”
